# Understanding the COVID-19 vaccine uptake, acceptance, and hesitancy in Ethiopia and Tanzania: a scoping review

**DOI:** 10.3389/fpubh.2024.1422673

**Published:** 2024-11-11

**Authors:** Esayas Kebede Gudina, Florida Joseph Muro, Norman Jonas Kyala, Tsegaye Melaku, Jane Brandt Sørensen, Dan Wolf Meyrowitsch, Zeleke Mekonnen, Tania Aase Dræbel

**Affiliations:** ^1^Institute of Health, Jimma University, Jimma, Ethiopia; ^2^Institute of Public Health, Kilimanjaro Christian Medical University College (KCMUCo), Moshi, Tanzania; ^3^Department of Community Medicine, Kilimanjaro Christian Medical Centre (KCMC), Moshi, Tanzania; ^4^Global Health Section, Department of Public Health, University of Copenhagen, Copenhagen, Denmark

**Keywords:** COVID-19, vaccine acceptance, "anti-vax", Ethiopia, Tanzania, Africa

## Abstract

**Background:**

The development and implementation of COVID-19 vaccines have been a breakthrough in controlling the pandemic. However, the vaccination coverage in most low-income countries remains very low due to critical vaccine shortage and profound hesitancy. In this scoping review, we aimed to assess COVID-19 vaccine uptake, acceptance, and hesitancy in Ethiopia and Tanzania.

**Methods:**

The search was made in PubMed, Scopus, Embase, and Web of Science. Only original research articles focusing on vaccine acceptance and hesitancy were included. The studies selected for a full read were analysed using a thematic analysis approach.

**Findings:**

A total of 76 articles were included in the study, with 74 of them coming from Ethiopia. The study found an increasing trend in vaccine uptake over time. However, there was also an increase in hesitancy and a decline in willingness to receive the vaccine. The willingness to receive the COVID-19 vaccine in Ethiopia ranged from 18.5 to 88%. The main reasons for "vaccine hesitancy" included fear of side effects, concerns about long-term safety, doubts about vaccine effectiveness, lack of information, vaccine fast-tracking, and religious beliefs. The study also found that younger individuals, females, and pregnant women were less willing to receive the vaccine. The adverse events reported among vaccinated individuals were mostly mild. Most of the studies operationalised vaccine acceptance-hesitancy as dichotomous variables. However, the historical, political, and socio-cultural context in which vaccine acceptance and hesitancy occur was not given any attention. While there is a good amount of data from Ethiopia describing patterns of vaccine acceptance and hesitancy among different populations over time, there is limited information from Tanzania due to the late arrival of the vaccine and limited published articles.

**Conclusion:**

We have observed a paradox involving two seemingly conflicting trends: an increase in vaccination rates/coverage and "anti-vax." Most studies have simplified vaccine acceptance-hesitancy as an “either-or” incident, without considering its dynamic nature and occurrence within a broader political, social, and cultural context. Therefore, it is crucial to explore approaches that can enhance our understanding of the vaccine acceptance-hesitancy phenomenon, in order to improve vaccine trust and uptake.

## Introduction

The COVID-19 pandemic has underscored the pivotal role of vaccination in combating a global health crisis. With compelling evidence demonstrating the effectiveness of COVID-19 vaccines in preventing severe illness (1, 2), reducing mortality (3), and curbing community transmission (4), the COVID-19 vaccines represented a beacon of hope. However, amidst these advances, an alarming disparity in vaccination rates has emerged, particularly in African countries (5). While regions such as Europe, North America, and South-East Asia have achieved impressive vaccination rates, with over 100 doses administered per 100 population, Sub-Saharan African (SSA) nations, including Ethiopia and Tanzania, have faced substantial challenges in attaining comparable coverage levels (6).

As of March 2023, an important milestone has been achieved, with 70.5% of the world’s population having received at least one dose of a COVID-19 vaccine. Impressively, a total of 13.5 billion doses have already been administered worldwide by then (6). However, these encouraging statistics mask disparities, particularly in low-income countries, where only 32.6% of the population has received at least one dose. In the specific context of Ethiopia and Tanzania, where vaccination rates stand at 42.5 and 52.6% respectively, understanding the determinants of COVID-19 vaccination uptake, acceptance, and hesitancy among both the public and healthcare providers is of paramount importance (6). We chose to explore the COVID-19 "anti-vax" vaccine hesitancy and acceptance in Ethiopia and Tanzania, as these countries represent two unique and contrasting approaches to the COVID-19 pandemic.

In addition to the shortage of vaccine supply in these settings, "anti-vax" has become a significant challenge (7). "Anti-vax" is defined as the delay or refusal to receive vaccines despite their availability (8, 9). While "anti-vax" has been present since the early days of vaccination, there has been a significant increase in vaccine-sceptical perceptions, narratives, rumours, and anti-vaccine movements in the past decade (10). Today, "anti-vax" is one of the most important barriers to preventing and controlling the spread of infectious diseases (11). This has become particularly evident with the worldwide availability of COVID-19 vaccines. Some countries have expressed concerns about the safety and effectiveness of these vaccines. This trend of "anti-vax" was observed in Ethiopia and Tanzania, where both the general public and politicians (12), as well as many healthcare workers (HCWs) had a negative attitude towards COVID-19 vaccination (13). While there is a substantial body of literature on "anti-vax" regarding the COVID-19 vaccine, there is a notable lack of reviews that comprehensively assess the existing knowledge and research specific to Ethiopia and Tanzania in this context.

"Anti-vax" is a social phenomenon that is influenced by various factors, such as time, place, and context (14). Some key determinants of "anti-vax" include mistrust in the vaccine’s safety and effectiveness (8), lack of trust and confidence in the healthcare system (15), affordability issues, and inadequate recognition of disease risk (8, 16). The financial cost of a vaccine also plays a significant role in determining willingness or intention to receive vaccines, particularly in low- and middle-income countries. Risk perception is another predictor of adult vaccination behaviour (17). Risk perception in health can be assessed through two dimensions: “*the perceived vulnerability or likelihood of harm if no action is taken and the perceived severity or seriousness of the consequences if harm were to occur*” (18). “These risks are weighed against the perceived costs and benefits of taking actions to prevent harm. Perceptions of risk can influence vaccine decision-making in two ways: perceived risks of vaccine-preventable diseases can promote vaccine acceptance, while perceived risks of vaccines can contribute to vaccine refusal” (14). Many studies have found that vaccine acceptance is higher among those with good knowledge and a favourable attitude towards COVID-19 vaccine. Yet, there is no direct causal relationship between knowledge about vaccination and its acceptance (14).

While several studies have been conducted in Ethiopia, there is limited knowledge regarding the factors contributing to vaccine acceptance and hesitancy in Tanzania. The purpose of this scoping review was to achieve the following objectives: 1. Map out peer-reviewed publications and analyse factors that influence COVID-19 vaccine acceptance and hesitancy in both Ethiopia and Tanzania; 2. Identify the methodological approaches used and identify research gaps to inform future studies.

## Methods

This scoping review was conducted in accordance with the approach developed by Peters et al. (19). This framework highlights how literature searches in scoping reviews should include Populations/participants, Concept and Context (PCC). To ensure clarity and rigour of the review process, we followed the steps outlined in the PCC framework. The detail of each step and what we did at each stage is explained below.

### Defining and aligning the objective/s and question/s

For this scoping review, a preliminary search was conducted in 2022 in PubMed to acquire a foundational knowledge of relevant literature within the study field using the following MeSH terms and syntax: (“COVID-19 Vaccines”[Mesh]) AND “Tanzania”[Mesh] and (“COVID-19 Vaccines”[Mesh]) AND “Ethiopia”[Mesh]. This foundational knowledge acquired was useful to define the objective to assess COVID-19 vaccine uptake, acceptance, and hesitancy in Ethiopia and Tanzania. Further, this search allowed us to define the following research questions for studies reporting COVID-19 vaccine uptake, acceptance, and hesitancy:

- What is the rate of COVID-19 vaccine uptake in Ethiopia and Tanzania?
- How is COVID-19 vaccine acceptance and hesitancy reported in Ethiopia and Tanzania, including study setting and type, methods, population, approaches, and perspectives represented.
- What are the reported factors determining COVID-19 vaccine acceptance and hesitancy in Ethiopia and Tanzania?

### Developing and aligning the inclusion criteria with the objective/s and question/s

Studies were considered for inclusion if they focused on COVID-19 vaccine acceptance, "anti-vax", or adverse effects of COVID-19 vaccines in Ethiopia and Tanzania. In order to maximise the inclusion of studies, we opted not to impose any restrictions related to study duration, the number of subjects recruited, study setting within the two countries, or follow-up duration. Additionally, studies involving both health care workers and communities of all age groups residing in Ethiopia and Tanzania were included. We included studies published in English only and studies were excluded if they were conference abstracts, case studies, reviews, commentaries, contained incomplete data, or lacked variables of interest. Non-English studies were excluded due to language proficiency constraints and the need for accurate analysis.

### Describing the planned approach to evidence searching, selection, data extraction, and presentation of the evidence

We used the Preferred Reporting Items for Systematic Reviews and Meta-Analysis (PRISMA) extension for Scoping Reviews to increase transparency and rigour in the scoping review (20).

### Searching for the evidence

Three researchers (EKG, TD, and FM) systematically searched four online databases including PubMed, Scopus, Embase, and Web of Science. We selected these databases based on their relevance and extensive coverage of public health, medical, and social science research to ensure a thorough review of relevant literature. This subsequent systematic search was carried out from January to April 2023. Studies published until April, 2023, were included.

To identify other relevant studies, we screened the references of the identified articles and existing systematic reviews. The Medical Subject Headings (MeSH)/controlled vocabularies and keywords were used to build a search strategy for each database. The detailed search strategies are outlined in the Supplementary Table S1.

### Selecting the evidence

**Figure 1:**
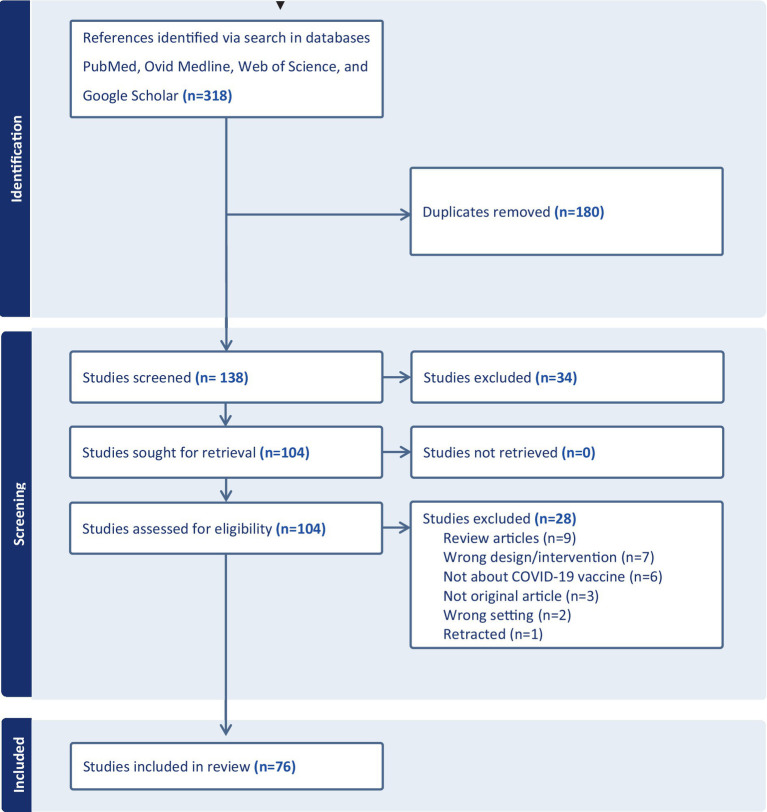
PRISMA flow diagram of study selection and inclusion process.

The initial literature search yielded 318 articles. After removing 180 duplicates, the titles and abstracts of remaining 138 studies were independently screened by EKG, TD, and FM using the online Covidence software package (21). After the abstract review, another 34 articles were excluded, leaving 104 articles that were retrieved for full-text review. During the full-text review, a total of 28 articles were excluded for the following reasons: Review articles (*n* = 9), wrong design/intervention (not focusing on COVID-19 vaccine acceptance, hesitancy, or side effects; *n* = 7), not related to the COVID-19 vaccine (*n* = 6), not an original research article (case report or commentary; *n* = 3), conducted in a setting other than Tanzania or Ethiopia (*n* = 2), and 1 article was excluded as it had been retracted after publication. Overall, 76 articles were included in the scoping review. Subsequently, the full texts of each potentially eligible article were reviewed to compile the final list of studies for analysis. Any disagreements were resolved through discussion among the three reviewers. The process of selecting and including studies is illustrated in Figure 1.

### Extracting the evidence

Data extraction was conducted using a data extraction sheet created within the Covidence software package. This process adhered to the guidelines for data extraction outlined in the PRISMA-ScR (20). Data pertaining to the study and participant characteristics, including information on first authors, country, publication year, journal, study setting (institution-based or community-based hospital-wide), sample size, study period, study design, results, conclusions, and recommendations were extracted (Supplementary data). To enhance rigor, we used multiple independent reviewers (EKG, TD, and FM) to ensure consistency. Differences between the reviewers were settled through discussion.

### Analysis of the evidence

The included studies were analysed using a qualitative and a quantitative approach. For the qualitative analysis, we used an inductive approach, which allowed for a more exploratory and open-ended analysis of the data. We opted for a thematic content analysis to identify emerging and cross-cutting themes from the included studies (22). Following this approach, categories and themes were not defined prior to the analysis, but rather emerged from the analysis. This approach allowed for more flexibility in the analysis. As a first step toward thematisation, the data were organised into 4 broad categories, which reflected each paper’s main focus. These categories were as follows: 1. Vaccine acceptance, uptake, and hesitancy; 2. Reason for "anti-vax"; 3. Factors associated with vaccine acceptance and hesitancy; 4. Vaccine side effects among vaccinated individuals. Following this step, we proceeded to a finer and more detailed reading of the data to identify “story-like” thematic units and then ordering these thematic units into cross-cutting themes/key concepts. Finally, we developed two models: 1. Illustrating the contextual continuum of vaccine acceptance and hesitancy; 2. the socio-cultural facets of vaccine acceptance and hesitancy.

### Presentation of the results

The results are presented in a narrative format, which is structured in relation to the aim of the scoping review and to answer the research questions. The narrative format is supplemented by figures and tables illustrating key characteristics of the studies included. The research team also developed a model which illustrates factors at play when people position themselves on the vaccine acceptance-hesitancy spectrum. In addition, the research team identified seven socio-cultural aspects which together offer a lens to capture what matters for people’s position on the spectrum of vaccine acceptance-hesitancy.

## Findings

### Characteristics of included studies

The included studies were conducted between 01 June 2020 and 15 July 2022 although nearly two-thirds of them (n = 47) were conducted after the COVID-19 vaccine arrived in Ethiopia in March 2021 (23) and Tanzania in July 2021 (24). Seventy-four of the studies were conducted in Ethiopia (25–98), while two of the included studies were from Tanzania (99, 100). Most of the studies (*n* = 71) were quantitative cross-sectional studies, while the rest used either mixed-methods (both qualitative and quantitative) (48, 57, 87) or qualitative design (81, 100). The sample size for cross-sectional studies varied from 191 (91) to 2,317 (83).

Twenty-eight studies involved HCWs, while studies involving community participants (*n* = 16), patients with chronic illness (*n* = 10), and pregnant women (*n* = 9) also contributed for a significant proportion of the included studies. In total, 53 studies were institution-based, 13 were conducted in the community, while 10 were online surveys (Table 1).

**Table 1:** Characteristics of included studies.

Study characteristics	Number of studies
Study participants	
Healthcare workers	28
Community (adult)	16
College/university students/teachers	11
Patients (chronic illness/HIV/cancer/outpatient visitors)	10
Pregnant women/lactating mothers	9
Others	2
Study design	
Quantitative cross-sectional	71
Qualitative	3
Mixed-methods (qualitative and quantitative)	2
Study setting	
Facility/institution-based	53
Community-based	13
Online/web-based survey	10
Timing	
Before vaccine arrival (until March 2021)	28
After vaccine arrival (since April 2021)	47
Unknown	1
Study sites	
Ethiopia	
Amhara Region (North Ethiopia)	27
Southern Nations and Nationalities Region	12
Nation-wide	11
Oromia	9
Addis Ababa	7
Eastern Ethiopia (Oromia, Harari, Dire Dawa)	6
Southwest Ethiopia Region	2
Tanzania	2

### Vaccination uptake/vaccination coverage increases over time

COVID-19 vaccination coverage/rate was reported as a proportion of participants who received at least one dose of the vaccines. Twelve studies reported data about COVID-19 vaccination rate (vaccine coverage/vaccine uptake); eight of these studies involved HCWs while two were conducted among community participants. Eleven studies reporting vaccination rate were from Ethiopia (27, 33, 39, 54, 56, 63, 80, 89, 90, 95, 96); there was one similar study from Tanzania (99).

In Ethiopia, the vaccine coverage among HCWs increased from 7.7% in April 2021 (90) to >50.0% from June 2021 (54, 63, 80, 89, 96). The overall pooled vaccination rate was 29.6% (95%CI: 28.7, 30.6), ranging from 14.4% (95%CI: 11.7, 17.1) among pregnant women to 46.3% (95%CI: 44.9, 47.7) among HCW (Supplementary Figure S1).

### Willingness to receive COVID-19 vaccine declines over time

**Figure 2:**
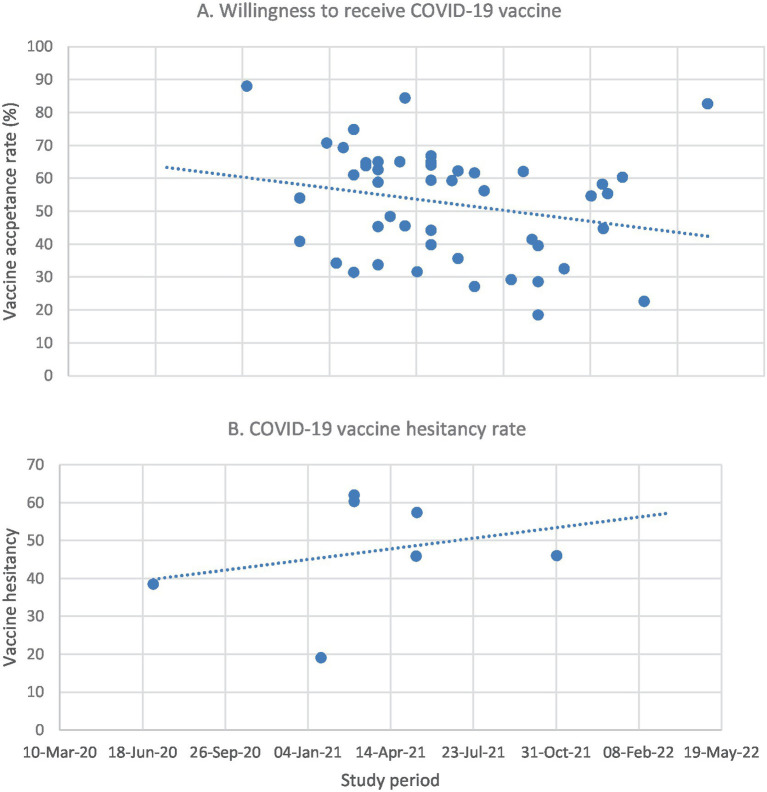
Vaccine acceptance **(A)** and hesitancy **(B)** over time in Ethiopia. No relevant data was available for Tanzania for this purpose.

Forty-eight studies reported data about COVID-19 vaccine acceptance. The related themes reported in individual studies presented in various forms such as vaccine acceptance, intention to receive vaccine/to be vaccinated, and willingness to receive vaccine/to be vaccinated. COVID-19 vaccine acceptance reported in the studies varied from 18.5% (48) to 88% (83). There is an overall trend of decline in willingness to receive COVID-19 vaccines over time (Figure 2A). While the reported vaccine acceptance rate was over 50% in most studies conducted until March 2021 (27, 45, 46, 64, 69, 72, 77, 79, 83, 85, 91, 94), five studies conducted since April 2021 reported rates of <30% (43, 48, 65, 87, 99).

In Ethiopia, the pooled COVID-19 vaccine acceptance rate was 58.5% (95%CI: 57.97, 59.04). The overall vaccine acceptance rate by regions of the study varied from 41.36% (95%CI: 39.57, 43.16) in Oromia to 74.52% (95%CI: 73.62, 75.42) in studies conducted across Ethiopia (Supplementary Figure S2). No data on acceptance rates and regional variations were found for Tanzania.

### Variations in vaccine acceptance and hesitancy vary in different study populations

Vaccine acceptance and hesitancy varied according to study populations. The pooled vaccine acceptance rate ranged from 46.0% (95%CI: 44.5, 47.5) among pregnant women to 65.2% (95%CI: 64.3, 66.1) in the community which is also slightly higher than that of HCW, 62.0% (95%CI: 60.9, 63.2; Supplementary Figure S3). On the other hand, "anti-vax" was found to be high among younger people (34, 51, 59, 75, 77, 99) and females (57, 77, 94). A study conducted in Tanzania by September 2021 among HCWs shows a vaccination rate of 18.5% (99). In Ethiopia, there was a trend of increasing vaccine refusal over time (Figure 2B).

### The identified studies approached vaccine acceptance and hesitancy as exclusive phenomena

Most of the studies operationalise vaccine acceptance-hesitancy as a dichotomy, isolate variables, which are related to either vaccine acceptance or "anti-vax" and serve as explanatory of the complex phenomena. Most identified studies tended to oversimplify and did not help improve the readers’ understanding of contextual conditions important for vaccine acceptance-hesitancy.

Certain knowledge and understandings of the COVID-19 vaccine was mentioned, however. For instance, pregnant women in Ethiopia were found to be hesitant towards the vaccination because they had knowledge about the unknown safety of the vaccine for their babies (27, 47, 86, 87). Another example is religious beliefs which when being isolated from the religious leaders’ positions cannot determine a person’s degree of vaccine acceptance or hesitancy. There was no mention of how knowledge or lack of knowledge about the effects of the COVID-19 vaccine can make the same person sway between vaccine acceptance and "anti-vax".

**Figure 3:**
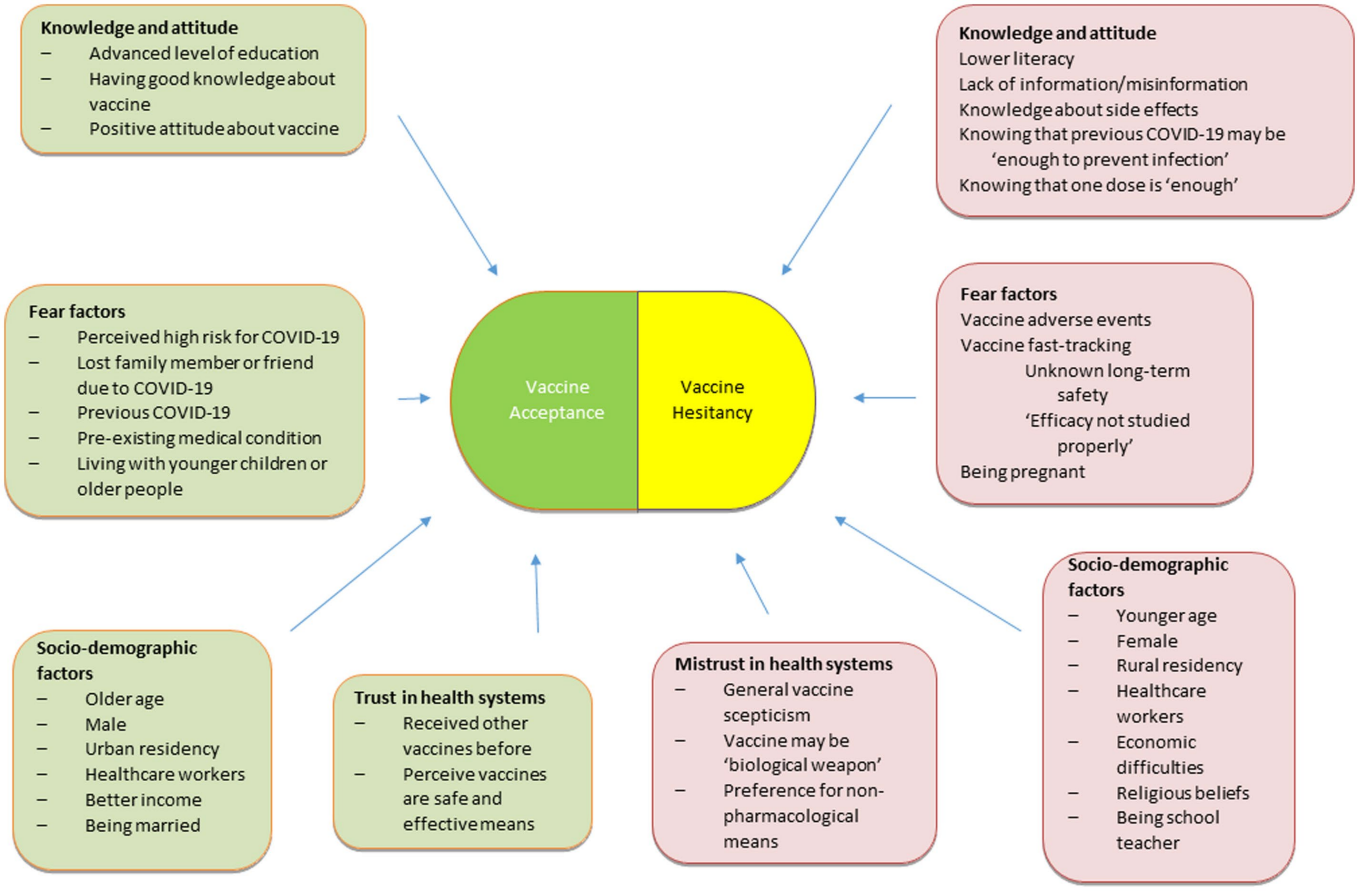
Vaccine acceptance-hesitancy spectrum.

Based on the findings from the included studies, we illustrated how certain variables may sway a person towards vaccine acceptance or hesitancy (Figure 3).

### Socio-cultural aspects of vaccine acceptance-hesitancy spectrum

Via a thematic analysis of the included studies, we identified seven socio-cultural aspects, which together offer a lens to capture what matters for people’s position on the spectrum of vaccine acceptance-hesitancy. The socio-cultural aspects were identified by coding the material and organising barriers and facilitators to COVID-19 vaccine into three large categories depending on whether they are related to health system, study population, community, or individual characteristics. Following this categorisation, a next analytical step consisted in finding themes and naming the themes according to their core characteristics. Together, the seven socio-cultural aspects form a model that provides insights into some of the core aspects at play in people’s consideration about the COVID-19 vaccination. These aspects are Adequacy, Affordability, Assurance, Acknowledgement, Adversity, Awareness, and Altruism.

### Adequacy of information

"Anti-vax" and refusal are attributable to inadequate information according to studies from Ethiopia (30, 33, 43, 44, 57, 59, 61, 81, 84, 87, 94, 98). Lack of adequate information may lead to beliefs that the vaccines may be biological weapons or intended to cause serious health hazards as reported by some of the studies (30, 33, 49, 57, 59, 80). When information fails to address doubts about vaccine effectiveness or uncertainties about potential risks of a vaccine, "anti-vax" and refusal may ensue (27, 30, 33, 39, 44, 46, 48, 49, 54, 57, 60, 62, 68, 76, 77, 80, 81, 84, 85, 87, 96–99).

Aynalem et al. (48) and Tefera et al. (87) demonstrate how inadequate information about benefits and risks related to COVID-19 vaccination result in "anti-vax" and refusal. This is particularly important among certain groups such as pregnant women who may hesitate or refuse the vaccine if their concerns about safety of their foetus are not addressed via adequate information. Indeed, these studies found that the lowest COVID-19 vaccine acceptance rate was reported among studies involving these groups where only 18.5% (48) and 22.6% (87) were willing to take COVID-19 vaccines. Concern for the safety of the foetus was the major reason for vaccine refusal (27, 47, 86, 87) among them. As a result, the COVID-19 vaccine coverage among pregnant women by March 2022, 1 year after the arrival of COVID-19 vaccines in Ethiopia, was only 14.4% (27).

### Assurances from healthcare provider and national health authorities

Assurance from health authorities and governmental policies are also predictors of vaccine willingness and acceptance. Mistrust in health authorities or lack of clarity, consistency, and transparency in governmental policies can also have cost in vaccine acceptance. Yamanis et al. (100) reported that the Tanzanian executive leadership change from a denialist president to a president who accepted vaccines and promoted transparency, global integration, easy vaccine access through multiple immunisation sites and free of charge vaccination, and community engagement and multi-sectoral collaboration has enhanced COVID-19 vaccination despite its late arrival in the country. However, the fact that the same people who were advocating against the vaccine are now leading the vaccination campaign and lack of clear communication from the government have led to mistrust and disinformation that contributed to the low vaccine acceptance. Besides, the limited human resource, lack of training, and the difficulty to reach remote areas were major limitations to ensure vaccine access and its fair distribution across the nation.

### Affordability

The large majority of the participants who intended to receive the vaccine were willing to receive it only if it was given for free and were less willing to pay for the vaccine (50, 70, 72, 73, 79, 83, 88). Studies with this focus showed that among the general population (79, 83), HCWs (73), and school teachers (70), the willingness to pay for the vaccine was moderate (79) or low (70, 73, 83). These studies showed that willingness to pay was influenced by perceptions of risk of being contaminated and benefits of the vaccine. Another study showed that among persons diagnosed with chronic conditions, the intention to get vaccinated is low (72). In this study, the intention to receive the vaccination was higher among persons with a health insurance, being retired, and with a higher socio-economic status (72).

In contrast to above studies, Strupat et al. showed that the willingness to pay for the COVID-19 vaccine was 88% among heads of households (83). The willingness to take the vaccine was “*significantly associated with COVID-19 cases in the family, trust in government and pro-social behaviour*” (83). Belsti et al. also found that vaccine acceptance is correlated to financial costs of the vaccine. Interestingly, Belsti et al. found a significant association between accepting the COVID-19 vaccine as the most important means to control the pandemic and believing that the vaccine should be distributed for free to the whole population (50). However, free of charge vaccination cannot stand alone without transparency, global integration, easy vaccine access through multiple immunisation sites, community engagement, and multi-sectoral collaboration, as shown by study from Tanzania by Yamanis et al. Moreover, mistrust in and disinformation by the people who recommend the vaccine heavily sway people’s decision away from vaccine acceptance (100).

### Acknowledgement of risks and benefits

Higher rates of vaccine acceptance were reported among older people (26, 27, 44, 46–48, 55, 62, 67, 72, 89, 91, 94), men (30, 37, 39, 41, 44, 68, 70, 71, 73, 76, 89, 93), and those with advanced level of education (25, 26, 31, 37, 39, 45, 49, 50, 61, 64, 69, 76, 87, 95) likely due to perceived high risk of COVID-19. Vaccine acceptance was found to be better among persons with pre-existing chronic medical conditions (40, 47, 48, 53, 58, 87, 93, 98), those with perceived risk of COVID-19 and risk of dying of it (33, 40, 44, 53, 60, 62, 64, 70, 73, 79, 88, 95, 96), those with previous COVID-19 (33, 49, 55, 56, 68, 71, 76), those who had family member or friend with COVID-19 or died of it (49, 52, 54, 76, 88, 96), and urban residents (45, 50, 71, 86).

"Anti-vax" and refusal were associated to people’s concerns about whether the vaccine was sufficiently tested for side effects prior to mass vaccination. Indeed, vaccine fast-tracking played into "anti-vax" (25, 30, 43, 44, 57, 62, 68, 80). Insufficient knowledge about the long-term safety of the vaccines was also put forward as an aspect at play in "anti-vax" and refusal (33, 43, 44, 54, 68, 76, 77, 81, 90, 94, 96–99). Some of the studies also showed that religious beliefs are reasons for "anti-vax" and refusal (25, 39, 48, 81, 86, 87, 96–98).

### Adverse effects

Fear of vaccine side effects was reported by most studies as a main reason for vaccine refusal (27, 30, 33, 39, 46, 48, 49, 54, 57, 59–62, 76, 77, 80, 84–87, 96–98). Ten studies, all conducted in Ethiopia, reported vaccine adverse effects among participants who received at least one dose of COVID-19 vaccines (27, 28, 38, 49, 66, 78, 80, 82, 92, 97). In all of these studies, the primary COVID-19 vaccine used was AstraZeneca/COVISHIELD (ChAdOx1); two of the studies also reported use of Johnson & Johnson (J&J) vaccine (Ad26.COV2.S) among some of the participants (28, 80). Among the studies that reported the overall prevalence of vaccine adverse effect, it ranged from 40% [28] to 96.3% (38). Injection site pain, reported by almost all the studies, was the most prominent of the vaccine related adverse events followed by fatigue, headache, and fever in decreasing order. Sleep disturbance and nightmares were also reported as important but less common side effects of the vaccine (80, 82).

Most of the symptoms of vaccine-related adverse events occurred within the first 24 h of vaccination (66, 78, 80, 82, 92, 97) and resolved within 3 days of onset (78, 80, 82). The adverse events resolved spontaneously for most while only about a third of the participants needed painkillers to relieve the symptoms (78, 80, 92, 97). Self-reported severe adverse events, defined as symptoms that led to interruption of daily activities or needed medical attention (66), were reported in less than 10% of the cases (78, 82, 97). However, adverse events needing hospitalisation were very rare (78, 80, 82). No study reported death or major sequelae after vaccination.

Adverse events were reported more frequently among younger individuals (<50 years) (28, 38, 66), female participants (28, 78, 82), and those with previous COVID-19 or COVID-19 like symptoms (78, 92). Concern for vaccine safety/fear of side effect (78, 80) and higher literacy (97) were also associated with reporting of side effects. Symptoms were more prominently reported in the first dose (78, 80, 92) than the second and in AZ than J&J (28).

Most of the participants who received the first dose of the vaccine were willing to receive the second dose and would likely recommend for others also (80, 82). However, participants who suffered from severe symptoms were less likely to receive the second dose (82). Other surveys involving cancer patients and HCWs reported that participants who received the first dose were hesitant to receive the subsequent dose because of side effects or discomfort after the first dose of the vaccine (33, 96, 97).

### Awareness

Awareness about who, where, and when one should be vaccinated and self-estimated sufficiency of information about vaccination or satisfaction with information on vaccination are frequently associated with vaccination decisions. This was also illustrated in this scoping review (26, 27, 30, 31, 33, 37, 39–41, 44–49, 52, 54–56, 58, 60, 62, 67, 70–72, 86–88, 91, 93, 95). and those with advanced level of education (25, 26, 31, 37, 39, 45, 49, 50, 61, 64, 69, 76, 87, 95). Intention to receive COVID-19 vaccine was also reported to be better among HCWs than other professionals (63, 85, 88). Among health professionals, physicians were more willing to receive the vaccine than other health professionals (40, 51, 75, 94). Among community participants, university teachers (79.7%) (32) and patients with chronic medical conditions (70.9%) (31) had a more receptive attitude towards the vaccine reflecting the importance health awareness on vaccine acceptance.

In Ethiopia, the level of awareness about COVID-19 vaccination was reported to be high (26, 29, 31, 32, 35, 36, 47, 74, 88, 91, 95). The composite level of knowledge about availability, benefit, effectiveness, safety, and vaccination strategy of COVID-19 ranged from 51.8% (91) to 93.8% (35) among HCWs and from 40.8% (74) to 73.6% (26) among community participants. However, the level of knowledge about the vaccination was not reflected in the level of vaccine acceptance. In spite of high levels of awareness, only about half of the HCWs had a favourable attitude towards the vaccine (29, 90, 91). Moreover, the pooled "anti-vax" (refusal) rate in Ethiopia was 45.05% (95%CI: 43.36, 46.75), being highest among HCW, 51.73% (95%CI: 49.41, 54.05) and lowest among general community participants, 34% (31.07, 36.93) (Supplementary Figure 3). Nevertheless, more than three-fourths of HCWs would recommend the vaccine for friends and family members (25, 35, 94).

### Altruism: for the benefits of others

An important predictor of decisions in vaccination is the subjective or social norm of a group. Dube et al. have demonstrated the importance of social aspects in vaccine acceptance and hesitancy. Vaccine acceptance is inked to a “*sense of social responsibility, or seeing vaccination as a duty of individuals in order to maintain herd immunity*” (14). Belsti et al. found that acceptance of vaccine is related to the idea that it is in the interest of the community. The opinions of family, relatives, friends, and colleagues matter for vaccine acceptance (50).

In other studies, vaccine uptake is linked to parents perceiving that their peers and family members are accepting the vaccine (101). Belsti et al. also found that the acceptance of the vaccine is related to the idea that there should be equal access to the vaccine (50). Strupat et al. have shown that willingness to take the vaccine is significantly associated with COVID-19 cases in the family, trust in government, and pro-social behaviour (83). If vaccine acceptance is a social norm of a community, the likelihood of a majority accepting vaccination is much higher. When in doubt, most people will look around and see to what the people around them and the people they respect do and if they are vaccinated and vaccinate their children, they will do likewise (102). Streefland and collaborators have shown that “*people have their children vaccinated because everybody does so and it seems the normal thing to do*” (15).

Although acknowledging being vaccinated can be of “benefit to others” may be linked to adult vaccination behaviour, it does not determine parents’ willingness to vaccinate their child. While some parents acknowledge that childhood immunisation contributes to “*building herd immunity*,” their decision to vaccinate was based on the perceived benefit to their own child rather than the “*benefits to others*” (103).

## Discussion

This scoping review aimed to assess COVID-19 vaccine uptake, acceptance, and hesitancy in Ethiopia and Tanzania. A large pool of local evidence on COVID-19 vaccine acceptance and hesitancy has been generated in Ethiopia. The studies, most of which were cross-sectional surveys, involved patients with chronic illnesses (diabetes mellitus, HIV, and cancer), pregnant women, HCWs, and other COVID-19 high-risk employees (teachers and bank workers). The COVID-19 vaccine acceptance rate in Ethiopia among both HCWs and the general community was lower than the global average and showed a declining trend after the arrival of the vaccine in the country. Younger individuals, females, and pregnant and lactating women were more hesitant to the vaccine. Fear of side effects, concern for long-term protection, lack of adequate information, lack of trust about vaccine efficacy, and religious beliefs were major reasons for "anti-vax". Although fear of side effects was the major reason for vaccine refusal, the reported vaccine adverse events in vaccinated individuals in Ethiopia were very mild and non-life threatening. On the other hand, only a limited number of published works were available from Tanzania, which may be a reflection of the government’s position on COVID-19 and its vaccine during the previous leadership.

Willingness to receive COVID-19 vaccine among the community and HCWs was reported to be relatively high during early 2021 in Ethiopia (27, 45, 46, 64, 69, 72, 77, 79, 83, 85, 91, 94). As a result, more than half of the HCWs received at least one dose of the vaccine within 3 months of its arrival in the country (54, 63, 80, 89, 96).

Acceptance of vaccine is highly correlated to healthcare professionals’ recommendations as shown in studies from other countries (104–106). A large study from the USA reported that the largest proportion of parents who changed their minds about delaying or not getting a vaccination for their children listed “information or assurances from healthcare provider” as the main reason (14). Trust in the persons who produce, distribute, or administer the vaccine is a predictor of vaccine acceptance. Brownlie and Howson studied trust and MMR vaccination and found that trust can be defined as a “a complex relational practice happening within particular socio-political context” (107). Trust is more than knowledge, a leap of faith, that persons may have based on the quality and strength of the relationship with health professionals (14). This could also explain that there was also a preference for vaccines developed in Europe and USA over those produced elsewhere (26, 77, 80). Interestingly, Belsti et al. found that healthcare professionals, even those who give the vaccine, are consistently hesitant towards vaccine (50).However, many studies conducted after the first vaccination campaign in Ethiopia showed declining trends in vaccine acceptance and increasing vaccine scepticism (43, 48, 65, 87, 99). Although this pattern may be due to geographic heterogeneity of the studies, it indicates a gradual decline in vaccine trust due to misinformation and probably as a result of global anti-vax-movement (108).

An important predictor of decisions in vaccination is the subjective or social norm of a group. Dube et al. have demonstrated the importance of social aspects in vaccine acceptance and hesitancy. Vaccine acceptance is inked to a “*Sense of social responsibility, or seeing vaccination as a duty of individuals in order to maintain herd immunity*” (14).

Fear of the vaccine side effects and concern for long-term effects, particularly among pregnant women and younger people, were mentioned as the main reasons for refusing vaccines (33, 49, 57, 59, 60, 62). However, 10 studies involving more than total of 5,000 study participants among both HCWs and communities who received at least one dose of the vaccines revealed that the reported adverse events were very mild with no reported fatality and major sequelae (66, 78, 80, 82, 92, 97). It is thus likely that the "anti-vax" was probably due to lack of adequate information or mis-information about the vaccines (30, 33, 43, 44, 57, 59, 61, 81, 84, 87, 94, 98). The development of the vaccines over short time (25, 30, 43, 44, 57, 62, 68, 80) and lack of reliable information for the long-term safety (33, 43, 44, 54, 68, 76, 77, 81, 90, 94, 96–99) might also have exaggerated fear of vaccine side effects and doubt about vaccines. The fact that Ethiopia faced three of its major COVID-19 outbreaks after the vaccine arrival (March–April 2021, August–October 2021, and December 2021–January 2022) [109] might also have led to low trust on the vaccines among the HCW and general public.

Vaccine acceptance rate reported in most of the included studies from Ethiopia is lower than the global average of 67.8% reported in Wang et al. (109) and 60.8% in Mengistu et al. (110). Similar with reports from previous meta-analyses from around the world, the vaccine acceptance declined gradually particularly after the vaccines became available in the country. The lower vaccine acceptance among females, pregnant and lactating women, and those younger than 50 years is also the same as the global data (109).

The low vaccine acceptance rate among young population and reproductive age women in particular is a cause for concern due to predominantly young population in the included countries and the rest of sub-Saharan Africa. The reluctance to pay for the vaccine (50, 70, 72, 73, 79, 83, 88) and preferences for vaccines produced in Europe and USA rather than other settings (26, 77, 80) are other noticeable challenges. Thus, besides availing the vaccines, misinformation and conspiracy beliefs should be proactively acted upon. Moreover, as religious beliefs were also mentioned as reasons for vaccine refusal (50, 70, 72, 73, 79, 83, 88, 100), it is highly relevant to involve religious and community leaders to tackle vaccine hesitance and improve its acceptance.

While data from Ethiopia have helped describe the patterns of vaccine acceptance and hesitancy among different populations over time, there is a paucity of information from Tanzania due to late arrival of the vaccine and limited published articles. Even 3 months after vaccines became available in the country, only less than a fifth of the HCWs in Tanzania have received the vaccine and only 28.6% confirmed their willingness to receive it in the future. The reasons given for "anti-vax" are the same as reported from Ethiopia (99). In Tanzania, COVID-19 denial and vaccine refusal by the late president Magufuli, lack of transparency after policy change later on, interferences and misinformation from religious leaders, and limited human resource made it difficult to intensify vaccination campaigns once the vaccine became accessible in the country (100).

Although the studies have sought to present vaccine acceptance and hesitancy in different populations along with their covariates, the historical, political, and social determinants of the vaccine acceptance-hesitancy spectrum were not adequately addressed. Most of the studies operationalised vaccine acceptance-hesitancy as dichotomous and isolate variables rather than a complex and dynamic phenomenon. As a result, they did not contribute much to the understanding of the contextual conditions which are important for vaccine acceptance and hesitancy.

Most studies sought to identify individual factors determining vaccine acceptance and hesitancy. The historical, political, and socio-cultural context in which the phenomena of vaccine acceptance and hesitancy take place is hardly given any attention. This is quite an important gap in knowledge, especially since the phenomenon of vaccine acceptance and hesitancy is complex and difficult to understand when dichotomised or operationalised as either/or. Individual decisions about vaccine acceptance-hesitancy are better understood as a complex of emotional, social, cultural, spiritual, political, and cognitive factors and cannot be understood in terms of “either/or” or when dichotomised. In reality, vaccine acceptance and hesitancy are better understood as a spectrum or a continuum. People’s position on vaccine acceptance and hesitancy is dynamic and subject to change according to contexts and circumstances. In addition, there is very little knowledge on the importance of public health, vaccine policy, the role of communication, and media and health professionals in vaccine acceptance and hesitancy in Ethiopia and Tanzania during the COVID-19 pandemic. Vaccine acceptance-hesitancy should ideally be treated as a spectrum and a continuum because people’s position on vaccine acceptance and hesitancy is dynamic and subject to change according to context and events (111). We identified some of the socio-cultural facets, which are important for people’s position on the spectrum of vaccine acceptance and hesitancy and developed a model that may be useful for understanding the phenomenon.

With the current trend of low vaccination rate and overwhelming hesitancy reported in the included studies, and evolution and global spread of new variants (112), vaccines remain the most viable means to combat the impact of the virus. Hence, a multifaceted approach is needed to bolster public confidence in the vaccines and to enhance vaccine coverage. We believe that the models developed based on our review may help to understand the vaccine acceptance-hesitance spectrum and their covariates. This may ultimately help in designing comprehensive strategies to deal with this phenomenon.

These models may help understand the vaccine-hesitancy phenomenon not just for COVID-19 but also for other types of vaccines. Furthermore, as the threat posed by new variants of SARS-CoV-2 is far from being over, strategies to enhance vaccine acceptance remain priority agendas. Others have argued that the threat posed by lack of vaccine access and shortages is a greater threat than "anti-vax" (113). It is thus essential to explore in-depth such social, behavioural, and political determinants to improve public trust on vaccine.

### Strengths and limitations

This review employed a comprehensive search of literature to map existing body of original research on uptake, acceptance, and hesitancy of COVID-19 vaccination in Ethiopia and Tanzania. Three reviewers carefully identified, read, selected and analysed the included studies. The analysis was an iterative process and qualified by the group of authors. A systematic review with critical appraisal could have provided us with other findings. However, there are three main concerns about this study: 1. The presence of significant variations in how concepts like knowledge and attitudes were defined and operationalised across the various studies included in the review. 2. The included studies might have missed to capture the more subtle determinants of vaccine uptake and hesitancy, and their combined effects. 3. Additionally, these surveys might only include questions that researchers assume are relevant, potentially overlooking important information. The studies are different in their approaches to the question of vaccine acceptance-hesitancy. The tone of the studies might have affected this. For instance, certain studies were interested to study just “"anti-vax" or refusal” while others focused on “vaccine acceptance.” The way the researchers structured their questions and asked participants is likely to bias the response.

Furthermore, the disparity in the number of studies from Ethiopia (74) compared to Tanzania (2) highlights challenges in data availability and may limit the ability to compare vaccine acceptance and hesitancy findings between the two countries.

## Conclusion

COVID-19 vaccine acceptance in Ethiopia and Tanzania among HCWs and communities was found to be lower than the global average and showed a declining trend over time, particularly in Ethiopia. Fear of side effects, concerns about long-term health impact and doubt about vaccine effectiveness were major reasons for "anti-vax." The reported vaccine side effects among vaccinated people were very mild and self-limiting conditions in most of the cases. Our scoping review showed a paradox of two seemingly conflicting trends of increase in vaccination rate/coverage and "anti-vax," a phenomenon beyond the scope of this review to explain. There is a gap in knowledge about the complexity of individual decision-making regarding vaccination. The importance of emotional, cultural, social, spiritual, and political factors as much as cognitive factors for individual decision-making were not also considered in the literatures.
