# Supplementary material for: Understanding the COVID-19 vaccine uptake, acceptance, and hesitancy in Ethiopia and Tanzania: a scoping review
Source: Front Public Health. 2024 Nov 11;12:1422673. doi: 10.3389/fpubh.2024.1422673 (PMC11586356; doi:10.3389/fpubh.2024.1422673)
Supplement: Supplementary file 1 [file Data_Sheet_1.docx]

# Understanding the COVID-19 vaccine Uptake, Acceptance, and Hesitancy in Ethiopia and Tanzania: A scoping review

*Esayas Kebede Gudina, Florida Joseph Muro, Norman Kyala Jonas, Tsegaye Melaku, Jane Brandt Sørensen, Dan Wolf Meyrowitsch, Zeleke Mekonnen, Tania Aase Dræbel*

## Supplementary material

**Table S1**: Detail search strategy for PubMed

| **Search Query** | **Sort by** | **Filters** | **Search details** | **Result** | **Time** |
| --- | --- | --- | --- | --- | --- |
| ("COVID-19 Vaccin*") AND (acceptance OR hesitance OR uptake) AND (Ethiopia OR Tanzania) AND ((2020/1/1:2023/4/30[pdat]) AND (english[Filter])) AND ((2020/1/1:2023/4/30[pdat]) AND (english[Filter])) AND ((2020/1/1:2023/4/30[pdat]) AND (english[Filter])) | Most Recent | English, from 2020/1/1 - 2023/4/30 | ("covid 19 vaccin*"[All Fields] AND ("accept"[All Fields] OR "acceptabilities"[All Fields] OR "acceptability"[All Fields] OR "acceptable"[All Fields] OR "acceptably"[All Fields] OR "acceptance"[All Fields] OR "acceptances"[All Fields] OR "acceptation"[All Fields] OR "accepted"[All Fields] OR "accepter"[All Fields] OR "accepters"[All Fields] OR "accepting"[All Fields] OR "accepts"[All Fields] OR ("hesitance"[All Fields] OR "hesitancies"[All Fields] OR "hesitancy"[All Fields] OR "hesitant"[All Fields] OR "hesitate"[All Fields] OR "hesitated"[All Fields] OR "hesitating"[All Fields] OR "hesitation"[All Fields] OR "hesitations"[All Fields]) OR ("uptake"[All Fields] OR "uptakes"[All Fields] OR "uptaking"[All Fields])) AND ("ethiopia"[MeSH Terms] OR "ethiopia"[All Fields] OR "ethiopia s"[All Fields] OR ("tanzania"[MeSH Terms] OR "tanzania"[All Fields] OR "tanzania s"[All Fields])) AND (2020/01/01:2023/04/30[Date - Publication] AND "english"[Language]) AND (2020/01/01:2023/04/30[Date - Publication] AND "english"[Language]) AND (2020/01/01:2023/04/30[Date - Publication] AND "english"[Language])) AND ((2020/1/1:2023/4/30[pdat]) AND (english[Filter])) | 142 | 09:10:08 |
| ("COVID-19 Vaccin* acceptance") AND (Ethiopia OR Tanzania) AND ((2020/1/1:2023/4/30[pdat]) AND (english[Filter])) | Most Recent | English, from 2020/1/1 - 2023/4/30 | ("covid 19 vaccin* acceptance"[All Fields] AND ("ethiopia"[MeSH Terms] OR "ethiopia"[All Fields] OR "ethiopia s"[All Fields] OR ("tanzania"[MeSH Terms] OR "tanzania"[All Fields] OR "tanzania s"[All Fields])) AND (2020/01/01:2023/04/30[Date - Publication] AND "english"[Language])) AND ((2020/1/1:2023/4/30[pdat]) AND (english[Filter])) | 52 | 09:07:54 |
| ("COVID-19 Vaccin*") AND (Ethiopia OR Tanzania) | Most Recent | English, from 2020/1/1 - 2023/4/30 | ("covid 19 vaccin*"[All Fields] AND ("ethiopia"[MeSH Terms] OR "ethiopia"[All Fields] OR "ethiopia s"[All Fields] OR ("tanzania"[MeSH Terms] OR "tanzania"[All Fields] OR "tanzania s"[All Fields]))) AND ((2020/1/1:2023/4/30[pdat]) AND (english[Filter])) | 233 | 08:51:00 |


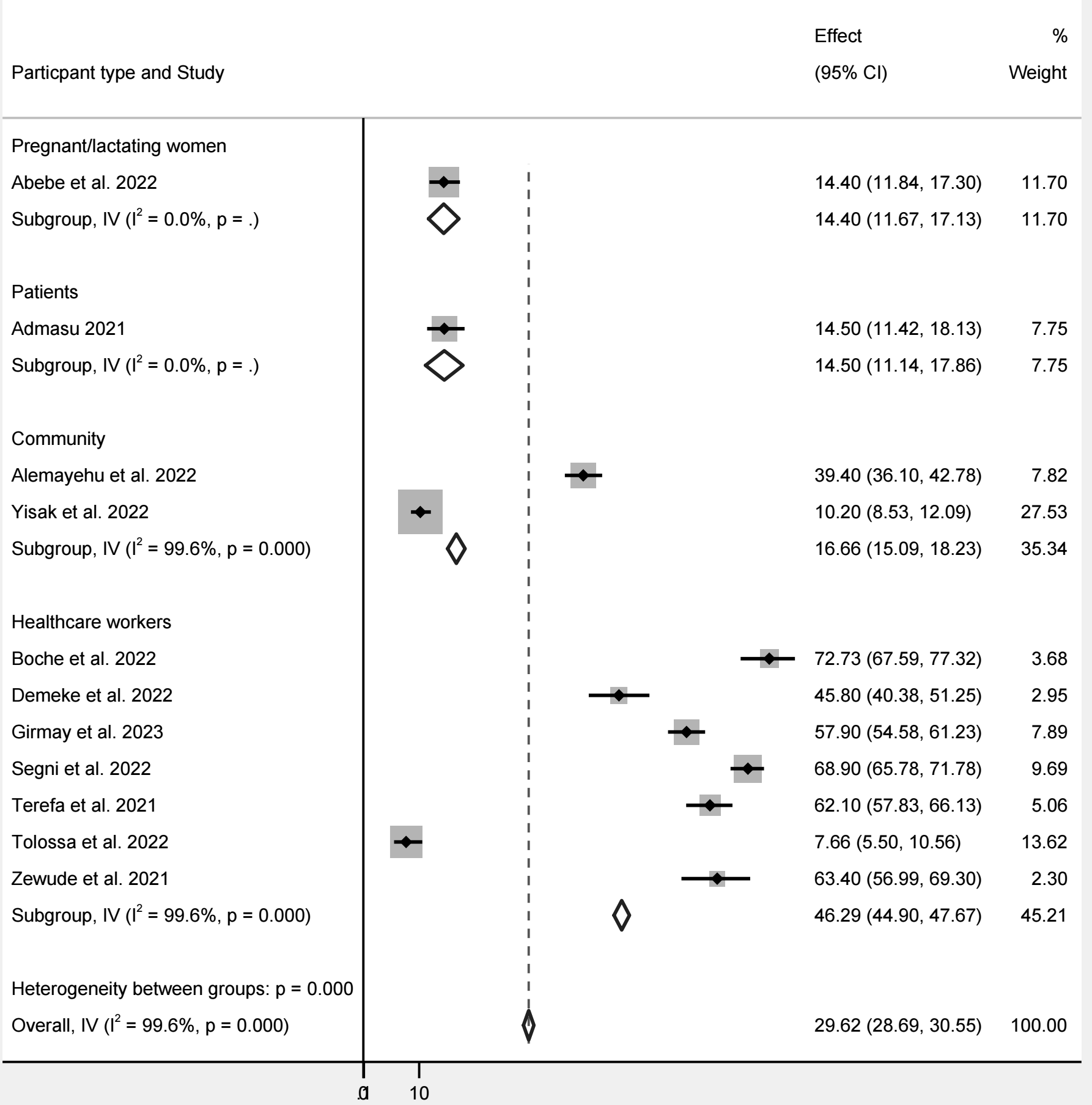


**Figure S1**: COVID-19 vaccination rate in Ethiopia


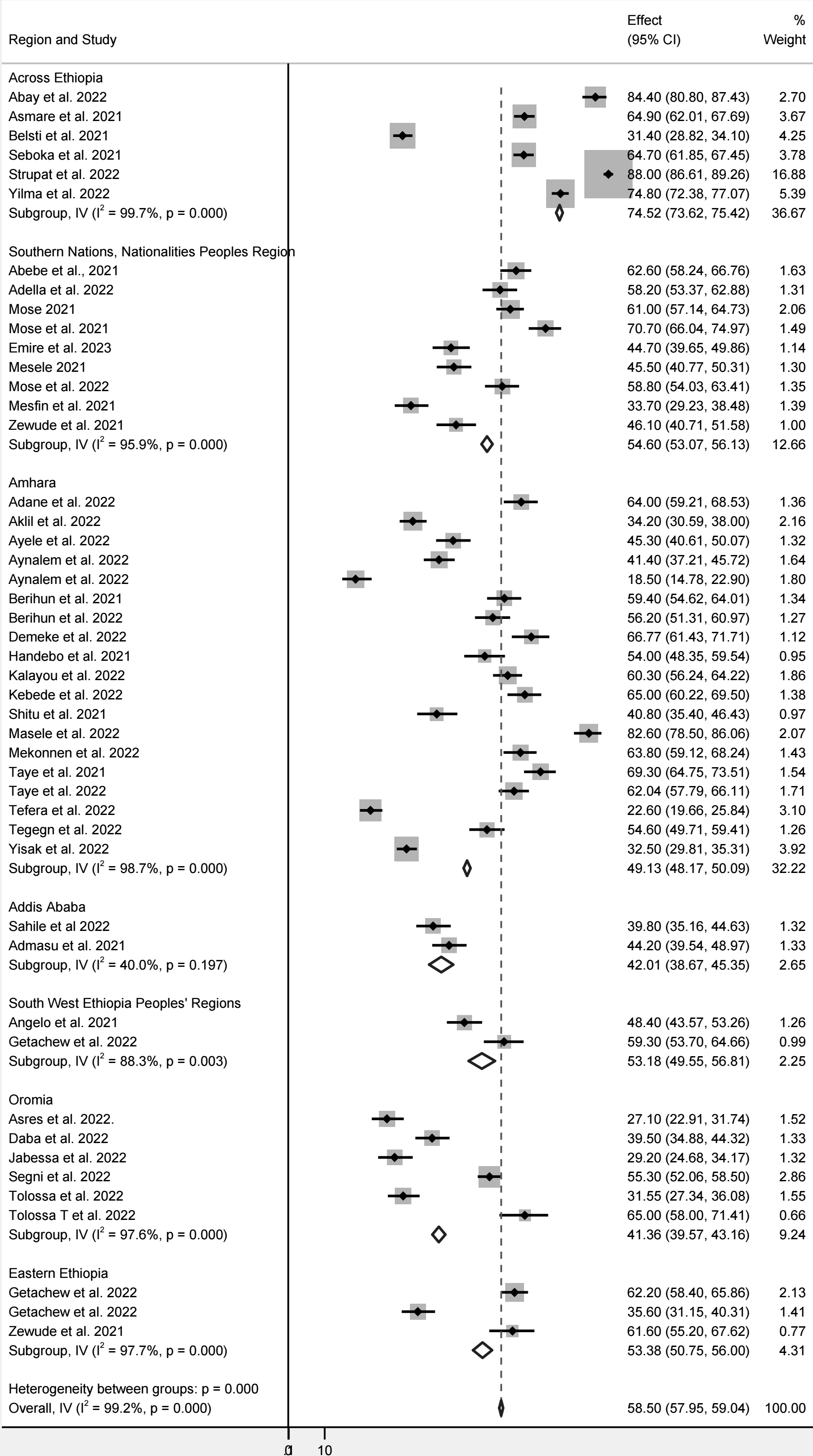


**Figure S2**: COVID-19 vaccination rate in Ethiopia by region of the study


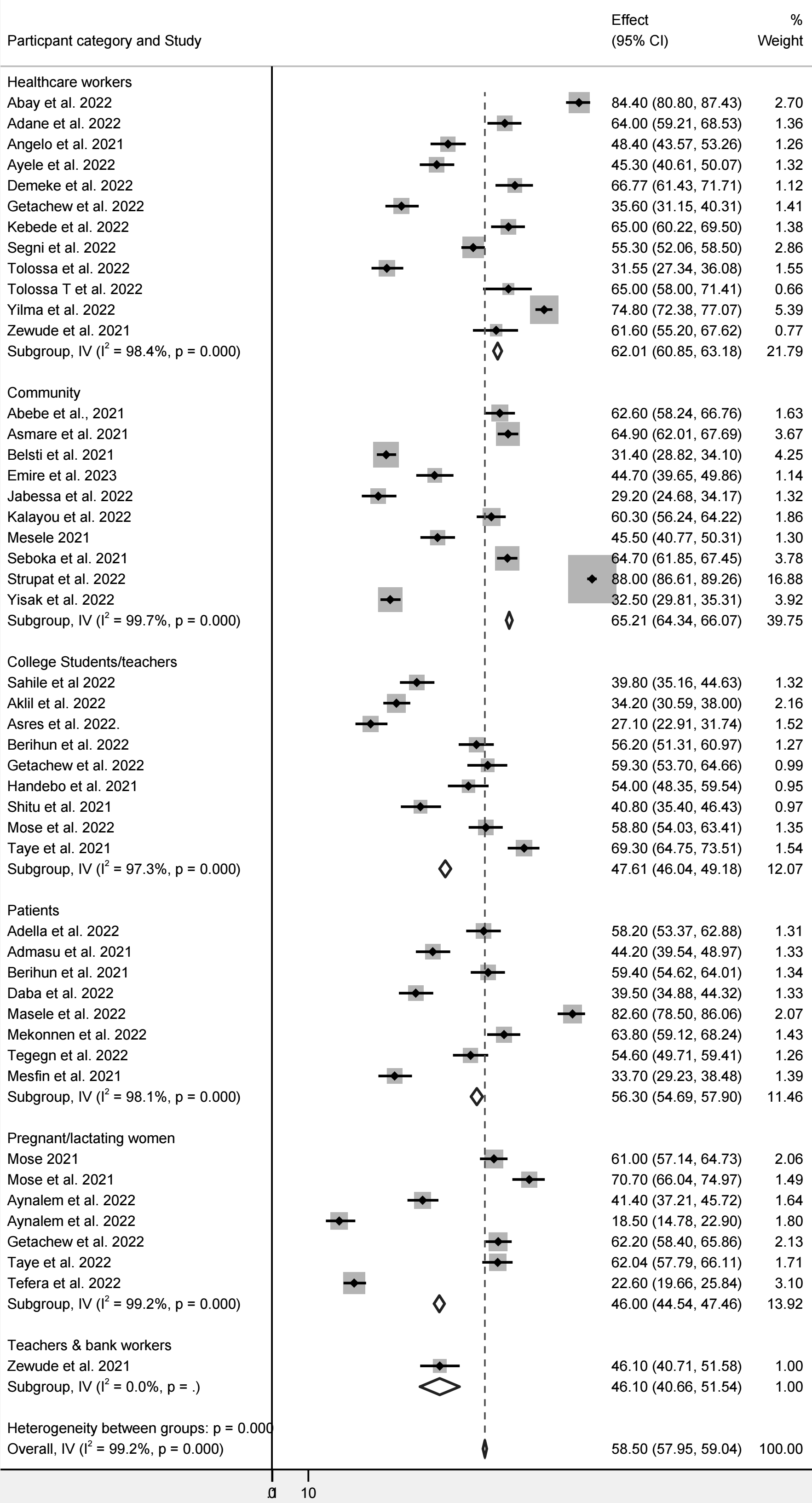


**Figure S3**: COVID-19 vaccination rate in Ethiopia by participant category
